# A New Optical Interferometric Biosensing System Enhanced with Nanoparticles for Alzheimer’s Disease in Serum

**DOI:** 10.3390/bios13070707

**Published:** 2023-07-05

**Authors:** Ana María M. Murillo, María Fe Laguna, Luis G. Valle, Luca Tramarin, Yolanda Ramirez, Álvaro Lavín, Beatriz Santamaría, Miguel Holgado

**Affiliations:** 1 Group of Optics, Photonics, and Biophotonics, Center for Biomedical Technology (CTB), Universidad Politécnica de Madrid, Parque Científico y Tecnológico de la UPM, Campus de Montegancedo, Pozuelo de Alarcón, 28223 Madrid, Spain; ana.martin@ctb.upm.es (A.M.M.M.); mariafe.laguna@upm.es (M.F.L.); luis.gvalle@upm.es (L.G.V.); luca.tramarin@ctb.upm.es (L.T.); yolanda.ramirez.alonso@gmail.com (Y.R.); alvaro.lavin@upm.es (Á.L.); beatriz.santamaria@upm.es (B.S.); 2 Group of Organ and Tissue on-a-Chip and In-Vitro Detection, Health Research Institute of the Hospital Clínico San Carlos, IdISSC, C/Profesor Martín Lagos s/n, 4ª Planta Sur, 28040 Madrid, Spain; 3 Department of Applied Physics and Materials Engineering, Escuela Técnica Superior de Ingenieros Industriales, Universidad Politécnica de Madrid, C/José Gutiérrez Abascal, 2, 28006 Madrid, Spain; 4 Department of Mechanics, Chemistry and Industrial Design Engineering, Escuela Superior de Ingeniería y Diseño Industrial, Universidad Politécnica de Madrid, Ronda de Valencia 3, 28012 Madrid, Spain

**Keywords:** Alzheimer’s disease, serum Tau protein, interferometry, optical biosensor, silanization, SiO_2_ nanoparticles

## Abstract

In this scientific work, we demonstrate, for the first time, a new biosensing system and procedure to measure specifically the total Tau (T-Tau) protein in serum, one of the most relevant biomarkers of Alzheimer’s disease (AD). AD is a progressive brain disorder that produces neuronal and cognitive dysfunction and affects a high percentage of people worldwide. For this reason, diagnosing AD at the earliest possible stage involves improving diagnostic systems. We report on the use of interferometric bio-transducers integrated with 65 microwells forming diagnostic KITs read-out by using the Interferometric Optical Detection Method (IODM). Moreover, biofunctionalized silicon dioxide (SiO_2_) nanoparticles (NPs) acting as interferometric enhancers of the bio-transducers signal allow for the improvement of both the optical read-out signal and its ability to work with less-invasive biological samples such as serum instead of cerebrospinal fluid (CSF). As a result, in this paper, we describe for the first time a relevant diagnostic alternative to detect Tau protein at demanding concentrations of 10 pg/mL or even better, opening the opportunity to be used for detecting other relevant AD-related biomarkers in serum, such as β-amyloid and phosphorylated Tau (P-Tau), neurofilaments, among others that can be considered relevant for AD.

## 1. Introduction

Alzheimer’s disease (AD) is the most common form of dementia, contributing to possibly 60–70% of prevalent neurodegenerative dementia cases [1,2]. AD is a progressive, irreversible, age-dependent, and neurodegenerative disorder with an insidious course that renders its pre-symptomatic diagnosis difficult [3,4,5]. The World Health Organization (WHO) declared dementia a priority condition through the Mental Health Gap Action Programme [6]. Currently, a total of about 36 million people in the world are estimated to suffer from this disease, which has a significant impact on the quality of life and independence of people. In fact, it is estimated that the incidence of AD will triple by 2050 due to, among other reasons, increased life expectancy and population growth [7].

AD is associated with neuronal dysfunction and death due to two neuropathological structures, β-amyloid (Aβ) plaques and neurofibrillary tangles (NFTs), formed by Aβ peptide aggregate and Tau protein, respectively. This leads to axonal degeneration, dysfunction of the cells involved, and loss of neuronal synapses, decreasing cognitive functions [1,8,9].

Currently, to diagnose AD, positron emission tomography, magnetic resonance imaging, and biomarkers in the cerebral spinal fluid (CSF) are used [8,10]. There are two biomarkers accepted for the early diagnosis of Alzheimer’s: the accumulation of β-amyloid (Aβ) in the brain, the low content in β-amyloid_1–42_ at the CSF, and the increase in the CSF of total Tau protein (T-Tau) or phosphorylated Tau (P-Tau) [11]. The use of both biomarkers (β-amyloid_1–42_ and Tau) at the same time has proven to be a worthy alternative for AD diagnosis, increasing sensitivity and specificity up to 90%. However, CSF is an invasive, complex, expensive, and painful biological sample to obtain, so it does not allow for easy monitoring of the disease in patients [12]. Moreover, it is reported that AD biomarkers are expressed in different body fluids [13]. Thus, the analysis of AD-related biomarkers in these non-invasive fluids will likely not only contribute to going a step further in the early detection of AD but also in monitoring the therapies to inhibit the aggregation of these proteins. Many studies propose serum-based biomarkers to facilitate clinical testing as a significant development in early diagnosis [8,12,14]. Additionally, these samples are significantly easier to obtain and much less invasive. However, it is a challenge to measure these related AD biomarkers in serum instead of CSF, such as Tau [10], mainly because the levels of these proteins are significantly much lower in serum than in CSF, and also it is a very complex matrix from which unspecific absorption must be avoided.

Tau protein is one of the serum-based biomarkers whose levels are associated with AD, being a predictive marker of the progression of neurodegeneration and making it possible to discriminate between patients who suffer AD and healthy people [1,7,10,11,14]. Tau is a Microtubule-Associated Protein (MAP) with a molecular mass of 78 kDa, whose function is to stabilize microtubules in neuronal axons, transport, signaling, and synaptic plasticity [11,12,14]. These functions are inhibited when Tau is phosphorylated [1]. Elevated concentrations of Tau protein in serum are associated with faster progression of the disease [8], suggesting that serum Tau measurements a highly relevant for the diagnosis of AD [12].

Moreover, there is an urgent and essential need to develop simple and reliable diagnostic tools capable of reaching the highest sensitivity and specificity, but at the same time, they can be rapid, cost-effective, and non-invasive to diagnose an AD treatment at an early stage when therapies are effective. It is also relevant to mention the importance of diagnostic methods to help health professionals make the best decisions since treatments are currently expensive.

Ultrasensitive technologies provide new opportunities for the development of serum-based biomarkers of AD pathology, such as a Single Molecule Array (SiMoA) [10] or an interdigitated microelectrode sensor system [15]. In addition, we also find different biosensors that are analytical devices for probing biomolecules by changing a biological response into electrical or optical signals for early diagnosis of AD, including Surface Plasmon Resonance-based biosensors (SPR) [16], electrochemical arrays [17], photoelectrochemical platforms [18], or field-effect transistor-based biosensors [19]. However, they have some limitations, such as non-specific binding, heterogeneity of surfaces, or not having simultaneous detection of different biomarkers [16,17,18,19]. Furthermore, to avoid the matrix effect and determine low concentrations of biomarkers, nanoparticles (NPs) are also a relevant alternative [20]. The advent of novel biosensor optic-based sensing methods contributes to a precise diagnosis.

In this paper, we demonstrate for the first time a competitive assay by employing an advanced optical In Vitro Detection (IVD) biosensing system to detect human Tau protein in serum and Phosphate Buffer Saline (PBS). This IVD system uses 65 wells, each with an interferometric bio-transducer, integrated into a single monolithic diagnostic KIT. Thus, this biosensing system is capable of performing 65 determinations, either to detect multiple biomarkers, one biomarker for multiple patients, or to increase the number of read-out repetitions to reduce measurement uncertainty. It is valuable to note that each well only requires one or two microliters of volume for each determination.

For this scientific work, a competitive assay was performed, which requires using the Tau protein as a bioreceptor onto the sensing surface of the bio-transducers. These bio-transducers are based on Fabry–Perot Interferometers (FPI), also known as Biophotonic Sensing Cells (BICELLs), which have been previously described in detail [21,22,23]. In addition, an example of FPI based on SU8 (a broadly used epoxy-based photoresist detailed in materials and methods) can be observed in previous publications [24,25], where it is reported how to anchor the corresponding bioreceptors. The read-out of the interferometric signal delivered for the bio-transducers is obtained using the Interferometric Optical Detection Method (IODM). This method to read out optical biosensors is based on the interferometric optical signals of two interferometers, one acts as a reference, and the other one is where the detection of biomolecules takes place, producing an Increased Relative Optical Power (IROP) [26,27]. The IODM has been validated and correlated with well-established analytical techniques that have been previously reported and demonstrated [25,28] for measurement in non-invasive real biological samples such as serum and saliva from patients and volunteers.

In particular, for this paper, the critical point was to achieve the demanding sensitivity needed to reach the required Limits of Detection (LoD) for the T-Tau as an AD biomarker in serum, avoiding the matrix effect. To this end, we employed silicon dioxide (SiO_2_) NPs, acting as interferometric enhancers of the signal produced by the FPI to reach the demanded LoD of this AD biomarker and to avoid the matrix effect of serum [18]. For this purpose, the αTau antibody is immobilized onto the SiO_2_ NPs. We call this conjugate αTau-NPs.

## 2. Materials and Methods

### 2.1. Diagnostic KIT and Read-Out Platform

Immunoassays were performed in diagnostic KITs of 65 wells. The size of each well is 1 mm in diameter, and the surface has an anti-reflective coating of SiO_2_. Located in the center of each well is integrated a SU8-based FPI with a circular shape of the sensing surface of 200 μm in diameter. This FPI produces the desired interferometric profile, as reported previously [25] in detail. 

The SU8 resist (MicroChem Corp., Newton, MA, USA) provides the epoxy groups necessary for protein immobilization. These epoxy groups, due to oxygen plasma activation (40 w, 45 s), open and leave the hydroxyl groups available to interact with the amino groups of the proteins given [24].

The read-out platform is based on the IODM, which is based on the optical interference signal produced for two interferometers, one for the reference and the other integrated with each of the wells forming the abovementioned diagnostic KIT. It is on the sensing surface of these FPIs where the corresponding bioreceptors are immobilized for the specific detection of the target proteins. The interferometric read-out signal is based on the IROP previously reported [22,27,29].

### 2.2. Biofunctionalization of SiO_2_ NPs with αTau Antibodies (αTau-NPs)

**Figure 1:**
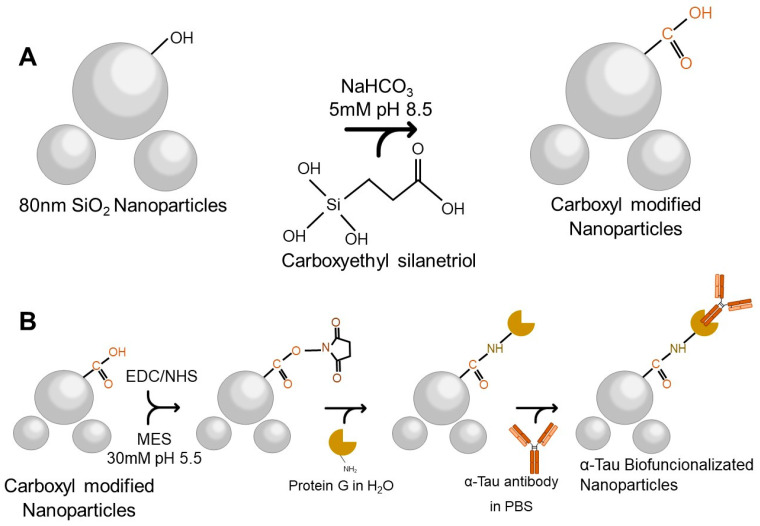
Biofunctionalization of SiO_2_ NPs with αTau antibodies. (**A**) Depiction of the silanization process of SiO_2_ NPs until the carboxyl functional group is obtained. (**B**) Representation of the final steps in the biofunctionalization process of the NPs where the G-protein and αTau antibodies are attached. Abbreviations: 1-Ethyl-3-(3-dimethylamino-propyl) carbodiimide (EDC), N-hydroxysuccinimide (NHS), 2-(N-morpholino) ethane sulfonic acid buffer (MES) and PBS.

As aforementioned, SiO_2_ NPs were conjugated with αTau antibodies through a silanization process, which modifies the surface for covalent αTau antibody immobilization [30] onto the SiO_2_ NPs by forming a silane layer. Firstly, a nominal concentration of 1 × 10^13^ NPs/mL of SiO_2_ NPs of 80 nm (Superior Silica, Chandler, AZ, USA) was washed three times with ultrapure water and diluted to a concentration of 1 × 10^10^ NPs/mL. Then, they were redispersed in a 5 mM sodium bicarbonate buffer pH 8.5 (NaHCO_3_) (Sigma-Aldrich, St. Louis, MO, USA) and 0.5 mM of carboxyethylsilanetriol (Fluorochem, Hadfield, UK) was added and incubated with shaking for one hour at room temperature (RT). Carboxyl group-modified NPs were obtained after centrifugation for 15 min at 7500 revolutions per minute (rpm) at RT. Pellets were washed three times with ultrapure water and stored at 4 °C (Figure 1A).

For the biofunctionalization process, it was necessary to activate the carboxyl group by adding 1-Ethyl-3-(3-dimethylamino-propyl) carbodiimide (EDC) 0.14 mM (Sigma-Aldrich) and N-hydroxysuccinimide (NHS) 1.2 mM (Sigma-Aldrich, St. Louis, MI, USA) in a 30 mM 2-(N-morpholino) ethane sulfonic acid buffer pH 5.5 (MES) (Sigma-Aldrich). This reaction was incubated with shaking for 60 min at RT. After three washes with ultrapure water, G-protein (Sigma-Aldrich) at a concentration of 50 ng/µL was added and incubated overnight at 4 °C with shaking in ultrapure water. To block the surface, diethanolamine 0.1 M (Sigma-Aldrich) was added and remained at RT for one hour with agitation.

Finally, three washes with ultrapure water were performed, and an αTau antibody (Sigma-Aldrich) at a concentration of 50 ng/µL was added and incubated overnight in agitation at 4 °C. After centrifugation for 15 min at 7500 rpm at RT, αTau-NPs were obtained (Figure 1B).

The hydrodynamic diameter and zeta potential of the NPs were measured by the Multi-Angle Dynamic Light Scattering (MADLS) method using the Zetasizer Ultra (Malvern, England) to characterize the biofunctionalization process. All measurements were carried out in triplicate at 25 °C.

### 2.3. Specificity Antibodies Assays

Affinity assays were performed to corroborate the proper functioning and non-cross reactivity of the antibodies and proteins used. Bovine Serum Albumin (BSA) (Sigma-Aldrich), Tau (Sigma-Aldrich), and p24 (human immunodeficiency virus capsid protein) (Abcam, Cambridge, UK) proteins were immobilized (2 µL at 50 ng/µL) in each well and recognized with their corresponding specific antibodies, αTau, αBSA (Sigma-Aldrich) and αp24 (Abcam), and the opposites.

### 2.4. Evaluation of the Assay Parameters

A curve response was performed to optimize the most suitable concentration of NPs for the IVD system. The initial read-out signal (without proteins immobilized) of each FPI of the KIT was obtained to control the immobilization process, and then, 2 µL of Tau protein at a concentration of 50 ng/µL was incubated inside the wells after the previous oxygen plasma activation of the FPI sensing surface. Diagnostic KITs were incubated in a wet chamber for 90 min at 37 °C and then washed with a 20 mL syringe with ultrapure water, followed by 45 s of shaking with ultrapure water and dried with dried and particle-less clean air.

After this process, the read-out signal confirms the correct immobilization of the Tau protein onto the sensing surface of the FPI transducers. Now at this stage, we call the functionalized FPI transducers with Tau, BICELLs (or bio-transducers) because they specifically detect the αTau antibody.

To obtain the biosensing response as a function of the αTau-NPs concentration, αTau-NPs were dropped in different wells of a KIT at different concentrations (from 1 × 10^4^ to 1 × 10^7^ NPs/µL) by dropping 2 µL per cell and incubated in a wet chamber for two hours at 37 °C. Finally, the KIT was washed with a 20 mL syringe of ultrapure water for 10 min in PBS pH 7.4 (Sigma-Aldrich) in shaking, 1 syringe of 20 mL of ultrapure water, and dried with clean particle-less air. Read-out IROP signal values were read out for monitoring the recognition read-out signal response of each αTau-NPs concentration.

αTau-NPs were also tested at a selected concentration of 10^6^ αTau-NPs in different wells of the diagnostic KIT biofunctionalized with Tau and BSA proteins (2 µL at 50 ng/µL in each well). The reason for this assay was to characterize its specificity. Incubation was performed at different times (1 and 2 h) in a humid chamber at 37 °C degrees to determine the optimal and necessary experimental time for subsequent assays. The washing step was performed with 20 mL of ultrapure water and shaking for 45 s in ultrapure water before drying with clean air.

### 2.5. Samples Preparation

PBS and Human Serum (Sigma-Aldrich) from platelet-poor human plasma and sterile-filtered were doped with different Tau protein concentrations (from 1 × 10^−2^ to 1 × 10^5^ ng/mL). Then, αTau-NPs conjugates were added to PBS or serum at 1 × 10^8^ NPs/µL, resulting in 1 × 10^7^ NPs/µL in the final mixture, and incubated for 1 h with shaking at RT. For the case of serum samples, we had to consider the Vroman effect, which describes the adsorption of serum proteins to a surface [31]. For this reason, different incubation times were tested, and finally, overnight at 4 °C exhibited good results. Finally, three washes with ultrapure water were performed and resuspended in PBS.

### 2.6. Tau Detection in PBS and Serum by Competitive Assay 

**Figure 2:**
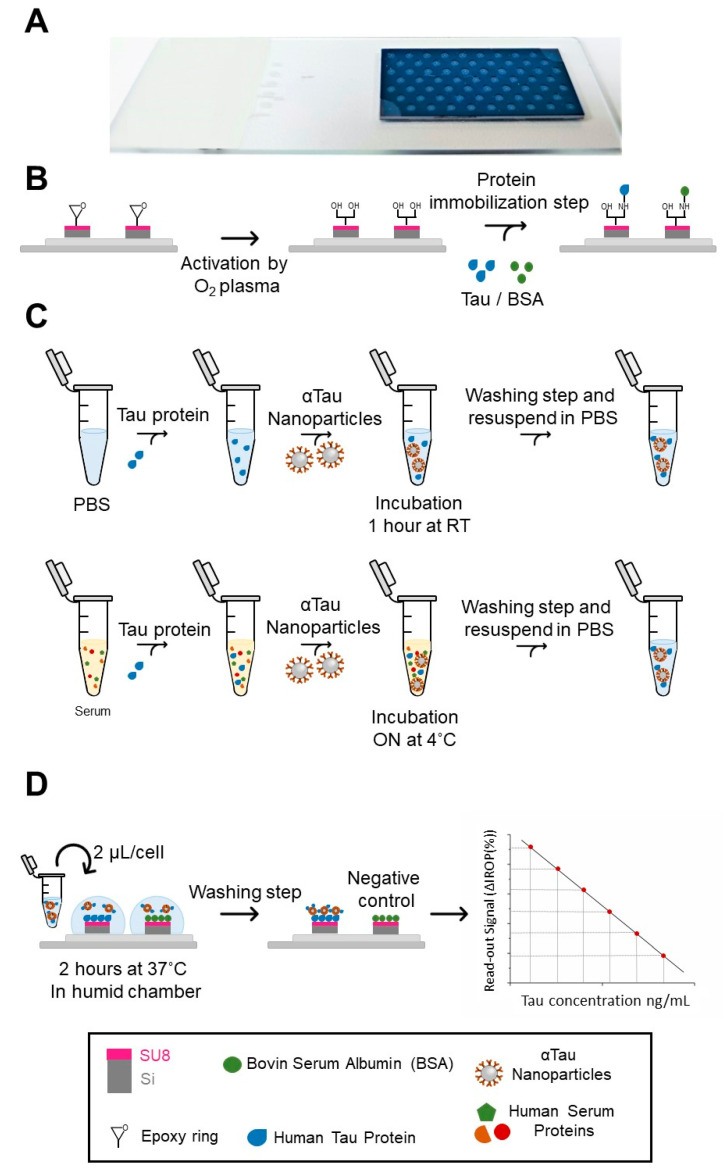
Outline of the protocol followed for the competitive assay. (**A**) Picture of 65 200-micron SU8 “BICELLs” diagnostic KIT used for the assays. (**B**) Process of protein immobilization on the sensing area of BICELLs by oxygen plasma activation. (**C**) Diagram of sample preparation in PBS and serum. (**D**) Depiction of the last steps of the competitive assay and the graph representing the inversely proportional relationship between the ΔIROP (%) signal and Tau concentration. Abbreviations: Biophotonic Sensing Cell (BICELL), phosphate buffer saline (PBS), and Increased Relative Optical Power (IROP).

To perform the competitive assay, first, plasma activation was applied on the sensing surface, and initial values of the KITs (Figure 2A) were read on the platform. Then, Tau protein was immobilized in the KITs (2 µL at 50 ng/µL), always having a negative control with immobilized BSA protein (2 µL at 50 ng/µL) under the same conditions as those above-described in Figure 2B. Secondly, the diagnostic KITs were blocked with casein hydrolysate 1× (Sigma-Aldrich) for 1 h under agitation at RT. 

Then, they were washed with a 20 mL syringe of ultrapure water, 2 min in PBS upon shaking, two syringes of 20 mL of ultrapure water, and dried with compressed air. For the recognition stage, αTau-NPs incubated in doped PBS with different concentrations of Tau protein (from 1 × 10^−2^ to 1 × 10^5^ ng/mL) (see Figure 2C) were deposited in the corresponding wells of the KIT and incubated for 2 h in a wet chamber at 37 °C. IROP values were obtained after each stage of incubation. Then, they were washed with a 20 mL syringe of ultrapure water, 10 min in PBS on shaking, two 20 mL syringes of ultrapure water, and dried with compressed air (Figure 2D). The same procedure was carried out for the competitive assay in a serum. Once the assay was performed in PBS, images were taken using Scanning Electron Microscopy (SEM) with a working distance of ≈10 mm, a high voltage of 5 kW, a probe current of 800 pA, and at normal incidence.

## 3. Results

In this work, we report for the first time how to detect the human Tau protein in PBS and serum by using IODM and diagnostic KITs consisting of 65 wells of 1 mm in diameter with integrated FPI-based bio-transducers of 200 μm in diameter per well just placed in the center of the well. 

**Figure 3:**
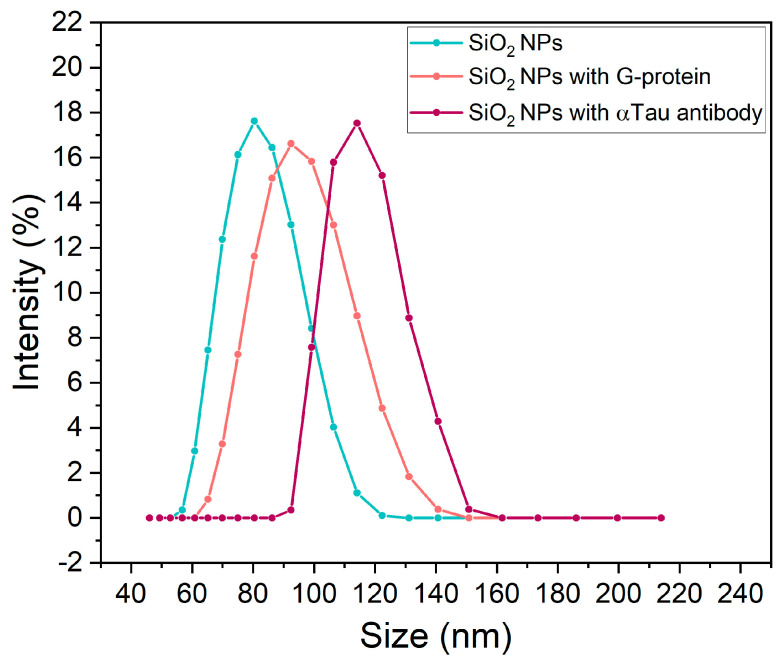
Size distribution of SiO_2_ NPs biofunctionalization process. Depiction of the increase in NPs size in the different steps of the biofunctionalization protocol. Abbreviations: Nanoparticles (NPs).

An applicable NPs biofunctionalization protocol was established for AD application. The surface modification was confirmed by zeta potential and hydrodynamic diameter measurements (Table 1). The SiO_2_ NPs starting material presented a negative zeta potential (in the range from −50 to −56 mV) consistent with a SiO_2_ surface, and the NPs have a diameter of 79.21 ± 2.7 nm according to the manufacturer’s specifications. After adding G-protein, the zeta potential increased to −40 ± 2.5 mV due to the positive charges of the amino groups of the G-protein, and the hydrodynamic diameter increased from 79.21 to 95.11 ± 1.3 nm. In the last step, after adding the antibody, the zeta potential decreased to −35 ± 0.4 mV, confirming the stability of the NP solution during the biofunctionalization process. In addition, the hydrodynamic diameter increased from 95.11 to 114.10 ± 2.8 nm (Figure 3), confirming the biofunctionalization of the NPs with the αTau antibody maintaining a negative charge on the surface; thus, the NPs are dispersed and stable in the aqueous solution.

We demonstrated the biofunctionalized αTau SiO_2_ NPs’ ability to capture Tau protein and their capability to be used in a competitive immuno-assay as optical enhancers to amplify the read-out signal to achieve the demanded LoD for this application.

**Figure 4:**
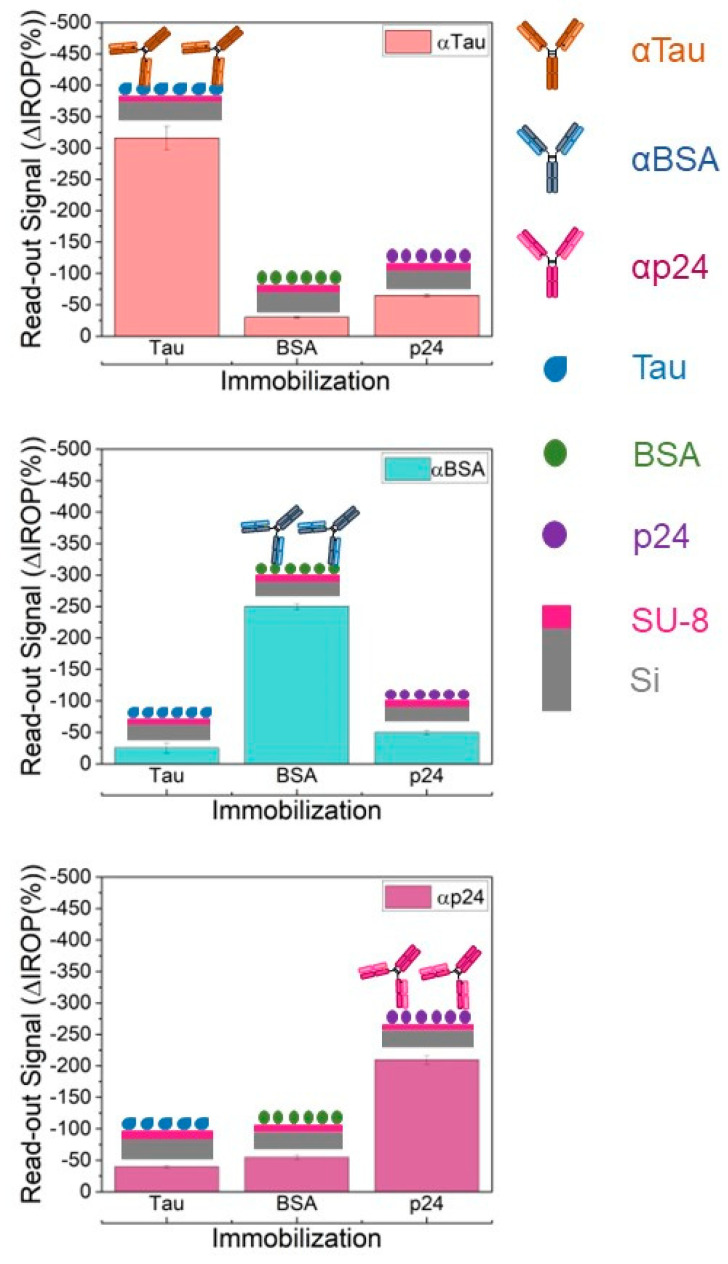
Specificity antibodies assays. The image above depicts the signal of the αTau antibody on the different proteins tested (Tau, BSA, and p24). The middle image indicates the signals of the αBSA antibody on these proteins, and the lower image corresponds to the signal of the αp24 antibody. Abbreviations: Increased Relative Optical Power (IROP) and Bovine Serum Albumin (BSA).

Several verification assays were also performed by immobilizing BSA, Tau, and p24 proteins to validate the specificity of the system and tested for cross-reactivity among the specific antibodies for these proteins (Figure 4). Therefore, the specificity was verified, although in all subsequent assays, only the BSA protein was used as a negative control.

**Figure 5:**
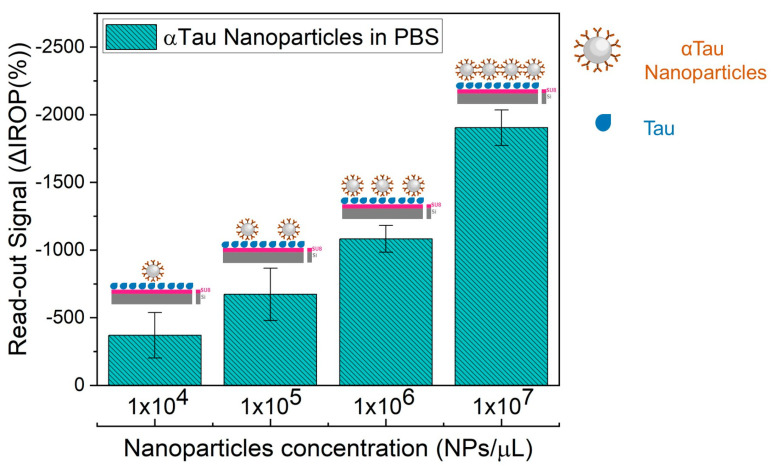
αTau nanoparticles calibration curve. Representation of the signal of the nanoparticles at different concentrations (1 × 10^4^ to 1 × 10^7^ NPs/µL) against immobilized Tau diagnostic KITs.

**Figure 6:**
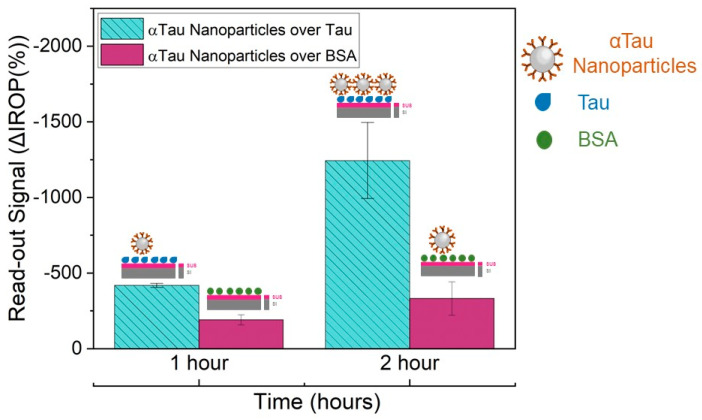
Specificity anti-Tau NPs assays. Recognition of αTau NPs over Tau and BSA at one and two hours of incubation. Abbreviations: Increased Relative Optical Power (IROP) and Bovine Serum Albumin (BSA).

The curve response of the IVD read-out signal as a function of the αTau-NPs allowed us to determine the right concentration αTau-NPs set at 1 × 10^7^ αTau-NPs/µL (Figure 5). We employed a single diagnostic KIT where αTau-NPs were incubated at different concentrations (from 1 × 10^4^ to 1 × 10^7^ NPs/µL) in several wells with its corresponding FPIs immobilized with Tau, as well as αTau-NPs on wells with its corresponding FPIs-immobilized BSA as a control during two hours at 37 °C in a humid chamber. Thus, we obtained the highest amplification of the signal covering the entire surface sensing and surface avoiding of the formation of bilayers. We also confirmed the functionality of the system as we were capable of obtaining a significant difference in the recognition signal specifically only over FPI biofunctionalized with Tau in contrast with the FPI functionalized with BSA used as a negative control. We also tested the appropriate incubation time for 1 × 10^7^ αTau-NPs/µL for one hour versus two hours. As a result, the incubation time was set at 2 h. This also confirms the specificity of the αTau-NPs since after two hours of incubation, we only obtained a signal against FPI immobilized with Tau protein (Figure 6).

Competitive assays were performed to determine low to high concentrations of Tau. Firstly, αTau-NPs at 1 × 10^7^ NPs/µL were tested in PBS. For this purpose, PBS was doped with different concentrations of Tau (from 1 × 10^−2^ to 1 × 10^5^ ng/mL) and then incubated with the αTau-NPs. These αTau-NPs were dropped in the FPIs with Tau immobilized. Five wells per concentration were used to reduce the uncertainty of the read-out signal. 

**Figure 7:**
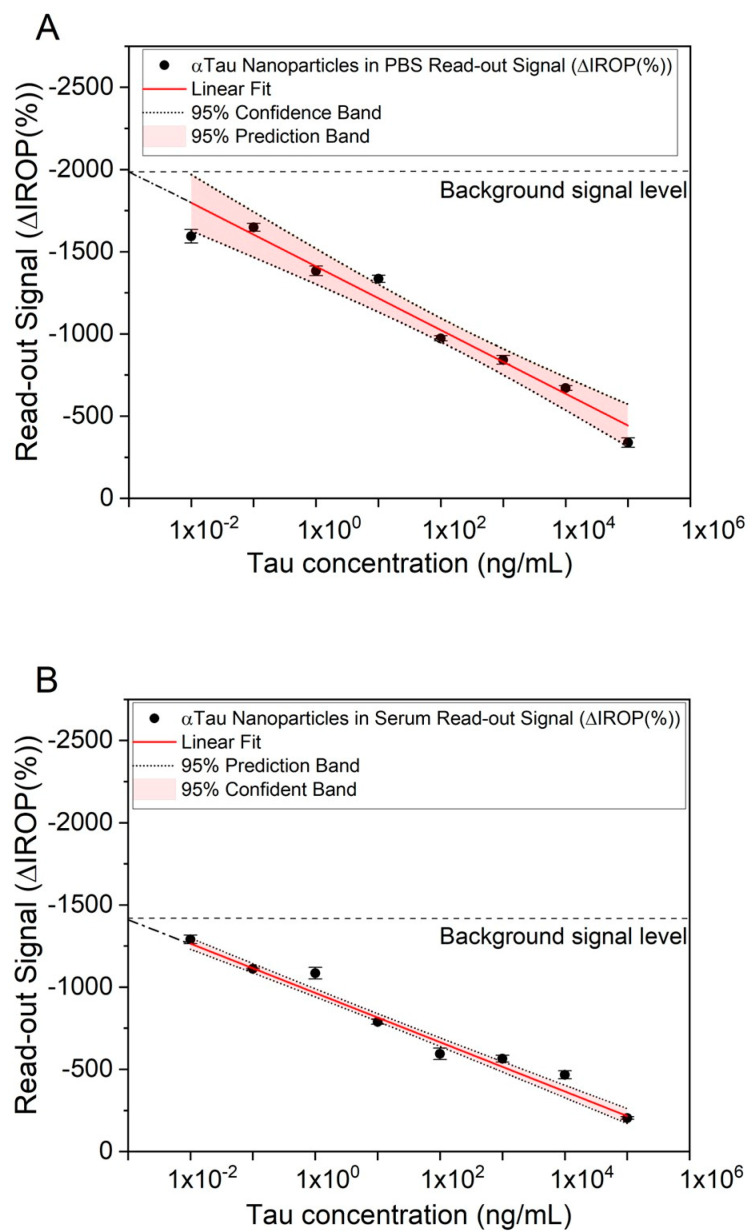
Competitive assays in PBS and serum. (**A**) Linear fitting between Tau concentration and ΔIROP (%) in PBS, with a 95% confidence interval and a 95% confidence band. (**B**) Linear fitting between Tau concentration and ΔIROP (%) in serum, with a 95% confidence interval and a 95% confidence band. The background signal level is plotted on the graph. Abbreviations: Increased Relative Optical Power (IROP) and Phosphate Buffer Saline (PBS).

We observed that low concentrations of Tau in the sample resulted in having more free antibodies on their surface, leading to a greater number of αTau-NPs bonded onto the sensing surface. In contrast, as the concentration of Tau increases, the αTau-NPs have fewer free binding sites, so fewer of them are recognized to the sensing surface of the FPI Tau immobilized. Finally, we obtained a curve response in PBS-correlating Tau concentration and ΔIROP (%) signal measurement (Figure 7A).

In the case of serum, the same experiment was performed, varying the incubation time, as we had to consider the Vroman effect mentioned above because proteins with higher mobility are attached to the NPs, which are then replaced by those with lower mobility but higher affinity, in this case by the antibodies that coat the NP [32]. It is important to remark here that these curves with PBS and human serum were carried out by repeating the whole assay on three different days. As a result, we obtained very similar signal levels, leading to good consistency and reliability of the biosensing responses during the different assays. In fact, the read-out signal represented is the average among these different assays, and the uncertainty considered is the standard deviation (see Figure 7). However, we also observed different signal levels for all the assays represented in the curve’s response between PBS (Figure 7A) and human serum (Figure 7B). We cannot ensure that the human serum may not have a basal concentration of T-Tau and that the serum sample matrix effect may play a role in this difference. Thus, the different signal level or signal offset observed between PBS and human serum was about 300 ΔIROP (%). 

A linear and inversely proportional correlation was obtained between the Tau concentration and the read-out ΔIROP (%) signal in human serum (see Figure 7B). We were able to read out a concentration of 10 pg/mL (see Figure 7) for both matrices (serum and PBS). However, we did not obtain the same read-out signal level, probably due to the nature and the different behavior of the matrices. 

**Figure 8:**
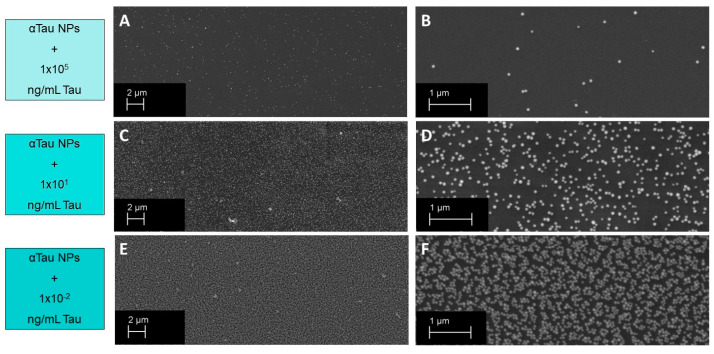
SEM images of competitive assays in PBS. (**A**) BICELL SEM image with a magnitude of 2K for a high concentration of Tau. (**B**) SEM image of the same BICELL with a magnitude of 10K. (**C**) BICELL SEM image with a magnitude of 2K for a medium concentration of Tau. (**D**) SEM image of the same BICELL with a magnitude of 10K. (**E**) BICELL SEM image with a magnitude of 2K for a low concentration of Tau. (**F**) SEM image of the same BICELL with a magnitude of 10K.

Finally, it is worth mentioning here the comparison between the curve response of the competitive immunoassay assay and the SEM images taken for different assay conditions (see Figure 7 and Figure 8). On the one hand, images were taken with the assay condition of a high concentration of Tau protein (10^5^ ng/mL) where the read-out signal level is very low, and the SEM micrographs show low αTau-NPs with a magnitude of 2K (Figure 8A) and 10K (Figure 8B). For the case of a Tau concentration of 1 ng/mL, a higher read-out signal and a higher number of αTau-NPs of the SEM micrographs onto the sensing surface can be clearly observed (Figure 8C,D). Finally, at the lowest Tau concentration considered (10^−2^ ng/mL), SEM images showed that the sensing surface is practically covered with αTau-NPs, resulting in the highest read-out sensing signal (see Figure 8E,F).

## 4. Discussion

A new biosensing system for early diagnosis of AD in serum is reported here based on IODM by using interferometric FPI-based bio-transducers of 200 μm in diameter of sensing surface integrated into microwells of 1 mm in diameter forming multiplexed diagnostic KITs by using SiO_2_ NPs acting as interferometric enhancers. 

This new diagnostic method proves to be specific for the protein of interest, avoiding cross-reactivity, increasing specificity, and increasing detection capability without the need for enzymatic chemical development.

A silanization protocol was established for SiO_2_ NPs and the biofunctionalization process with the antibody of interest for this application (αTau), obtaining specific nanoconjugates (αTau-NPs) for the sensing system.

This proposed silanization process is efficiently performed by anchoring the silane, minimizing chemical reaction steps. Furthermore, the use of EDC/NHS reagents opens up the possibility of anchoring other types of proteins in addition to G-protein, such as streptavidin, in order to bind biotin-labeled molecules (antibodies or antigens).

Thus, the proposed methodology is very versatile in carrying out determinations of different biomarkers. This versatility is also offered by the type of immobilization process on the sensor since, due to the activation of the sensor surface by using oxygen plasma, any molecule of interest can be covalently anchored [33,34].

We observed that the limit of detection reached with our system was in the order of 10 pg/mL (see Figure 7) with a dynamic range from 1 × 10^5^ ng/mL to 1 × 10^−2^ ng/mL. Thus, these figures fall within the reported detection range for being used in Tau concentration as an AD biomarker in serum because abnormal levels of Tau protein are reported as values higher than 450 pg/mL in CSF [32] or more than 30 pg/mL in serum [35]. Similarly, for p-Tau protein, levels are reported as those greater than 60 pg/mL in CSF [32] or 20 pg/mL in the case of serum [36]. These results also suggest that the IVD system reported in this article could be used for early detection of AD because the development methodology is competitive with other previously described biosensors and equals or improves the LoD of other optical sensors, such as biolayer interferometry [37] or surface plasmon resonance [38], as well as for electrochemical and potentiometric sensors (see Table S1 in Supporting Information).

This system equals or improves other related systems reported in the scientific literature. In addition to the low sample volume used, this methodology has other advantages, such as lower costs per determination, simplicity of handling, and being label-free, as no chemical developers are needed to obtain the signal. The sensitivity comparison of this work with other methods is shown in Table S1 of the Supplementary Material, in which it can be appreciated that this system has a lower detection limit than most of the biosensors in the overview. On the other hand, the LSPR-based biosensor [38] does have a lower detection limit but has only been demonstrated in PBS and not in real samples. Moreover, the sensitivity of this system can be improved depending on the concentration of the NPs used, and this optimization can be very interesting work in the future.

It is also worth mentioning that the diagnostic KITs reported in this article (see Figure 2A) use only 1–2 µL for each well in contrast with the hundreds of mL needed for ELISA well plates. Moreover, these diagnostic KITs enable us to perform multiplexed analysis with 65 determinations per KIT in a rapid and highly sensitive manner, either to perform multiple repetitions, to measure multiple concentrations, to measure other representative proteins of the AD, or simply to have a high screening capacity.

The immunoassay enhanced with NPs, not only significantly improves the sensitivity but has also been demonstrated to be used for a complex matrix such as serum, which is less invasive to obtain, opening up the possibility of a new, cost-effective, high-throughput technique for early AD detection. The reason remains in the use of nanoparticles acting as enhancers of the interferometric bio-transducers employing the IODM, allowing us to determine low concentrations of Tau protein in serum samples.

Finally, it is also relevant to highlight that serum samples are much less invasive than CSF samples, and for this reason, we believe that this method is a step further to anticipating diagnosis by measuring low concentrations of this protein in serum before the onset of clear symptoms of the disease. In addition, our achieved detection limit has been able to improve the capability of several of the systems proposed in the literature regardless of sensor type [31,32,36].

In this work, a diagnostic system enhanced with nanoparticles for Alzheimer’s disease in serum has been reported and optimized. However, certain limitations must be considered, as the concentration of nanoparticles and their relationship with the sensing surface must be carefully controlled. They must be well-characterized during the biofunctionalization process in order to avoid using nanoparticles that form aggregates. The behavior of these nanoparticles should be studied in other types of real samples or to measure other biomarkers. On the other hand, the detection limit levels should be further improved for those biomarkers that are found in lower concentrations. The following steps will be to improve even more the detection limit of the system and to extend the system for measuring other AD biomarkers in a single KIT and in other types of matrixes, such as tears or saliva. In this case, the protocols will have to be modified according to these requirements.

## Figures and Tables

**Table 1:** Zeta potential and hydrodynamic diameter measurements by MADLS of the SiO_2_ nanoparticles biofunctionalization process.

	SiO_2_ NPs	SiO_2_ NPs with G-Protein	SiO_2_ NPs with αtau Antibody
Zeta potential (mV)	−50.71 ± 4.38	−40.16 ± 2.50	−35.62 ± 0.40
Hydrodynamic diameter (nm)	79.21 ± 2.70	95.11 ± 1.30	114.10 ± 2.80

## Data Availability

Not applicable.

## Supplementary Materials

The following supporting information can be downloaded at: https://www.mdpi.com/article/10.3390/bios13070707/s1, Table S1: A comparison between different biosensor techniques for tau detection [39,40,41,42,43,44,45].

## References

[1] Lashley T., Schott J.M., Weston P., Murray C.E., Wellington H., Keshavan A., Foti S.C., Foiani M., Toombs J., Rohrer J.D. (2018). Molecular Biomarkers of Alzheimer’s Disease: Progress and Prospects. DMM Dis. Model. Mech..

[2] Jamshidnejad-Tosaramandani T., Kashanian S., Babaei M., Al-Sabri M.H., Schiöth H.B. (2021). The Potential Effect of Insulin on AChE and Its Interactions with Rivastigmine In Vitro. Pharmaceuticals.

[3] Andreasen N., Blennow K. (2005). CSF Biomarkers for Mild Cognitive Impairment and Early Alzheimer’s Disease. Clin. Neurol. Neurosurg..

[4] Sevigny J., Chiao P., Bussière T., Weinreb P.H., Williams L., Maier M., Dunstan R., Salloway S., Chen T., Ling Y. (2016). The Antibody Aducanumab Reduces Aβ Plaques in Alzheimer’s Disease. Nature.

[5] Jamshidnejad-Tosaramandani T., Kashanian S., Al-Sabri M.H., Kročianová D., Clemensson L.E., Gentreau M., Schiöth H.B. (2022). Statins and cognition: Modifying factors and possible underlying mechanisms. Front. Aging Neurosci..

[6] WHO (2012). Dementia: A Public Health Priority.

[7] Rabbito A., Dulewicz M., Kulczyńska-Przybik A., Mroczko B. (2020). Biochemical Markers in Alzheimer Disease. Int. J. Mol. Sci..

[8] Zetterberg H., Burnham S.C. (2019). Blood-Based Molecular Biomarkers for Alzheimer’s Disease. Mol. Brain.

[9] Michalicova A., Majerova P., Kovac A. (2020). Tau Protein and Its Role in Blood–Brain Barrier Dysfunction. Front. Mol. Neurosci..

[10] Li D., Mielke M.M. (2019). An Update on Blood-Based Markers of Alzheimer’s Disease Using the SiMoA Platform. Neurol. Ther..

[11] Mroczko B., Groblewska M., Litman-Zawadzka A. (2019). The Role of Protein Misfolding and Tau Oligomers (TauOs) in Alzheimer’s Disease (AD). Int. J. Mol. Sci..

[12] Nam E., Lee Y.B., Moon C., Chang K.A. (2020). Serum Tau Proteins as Potential Biomarkers for the Assessment of Alzheimer’s Disease Progression. Int. J. Mol. Sci..

[13] Lönneborg A. (2008). Biomarkers for Alzheimer Disease in Cerebrospinal Fluid, Urine, and Blood. Mol. Diagn. Ther..

[14] Olsson B., Lautner R., Andreasson U., Öhrfelt A., Portelius E., Bjerke M., Hölttä M., Rosén C., Olsson C., Strobel G. (2016). CSF and Blood Biomarkers for the Diagnosis of Alzheimer’s Disease: A Systematic Review and Meta-Analysis. Lancet Neurol..

[15] Kim Y.S., Yoo Y.K., Kim H.Y., Roh J.H., Kim J., Baek S., Lee J.C., Kim H.J., Chae M.S., Jeong D. (2019). Comparative Analyses of Plasma Amyloid-b Levels in Heterogeneous and Monomerized States by Interdigitated Microelectrode Sensor System. Sci. Adv..

[16] Rezabakhsh A., Rahbarghazi R., Fathi F. (2020). Surface Plasmon Resonance Biosensors for Detection of Alzheimer’s Biomarkers; an Effective Step in Early and Accurate Diagnosis. Biosens. Bioelectron..

[17] Song Y., Xu T., Zhu Q., Zhang X. (2020). Integrated Individually Electrochemical Array for Simultaneously Detecting Multiple Alzheimer’s Biomarkers. Biosens. Bioelectron..

[18] Kim K., Park C.B. (2020). Femtomolar Sensing of Alzheimer’s Tau Proteins by Water Oxidation-Coupled Photoelectrochemical Platform. Biosens. Bioelectron..

[19] García-Chamé M.Á., Gutiérrez-Sanz Ó., Ercan-Herbst E., Haustein N., Filipiak M.S., Ehrnhöfer D.E., Tarasov A. (2020). A Transistor-Based Label-Free Immunosensor for Rapid Detection of Tau Protein. Biosens. Bioelectron..

[20] Espinosa R.L., Garrido-Arandia M., Romero-Sahagun A., Herreros P., Tramarin L., Laguna M.F., Díaz-Perales A., Holgado M. (2020). A New Optical Interferometric-Based in Vitro Detection System for the Specific IgE Detection in Serum of the Main Peach Allergen. Biosens. Bioelectron..

[21] Lavín Á., Casquel R., Sanza F.J., Laguna M.F., Holgado M. (2013). Efficient Design and Optimization of Bio-Photonic Sensing Cells (BICELLs) for Label Free Biosensing. Sens. Actuators B Chem..

[22] Holgado M., Sanza F.J., López A., Lavín Á., Casquel R., Laguna M.F. (2014). Description of an Advantageous Optical Label-Free Biosensing Interferometric Read-out Method to Measure Biological Species. Sensors.

[23] Sanza F.J., Holgado M., Ortega F.J., Casquel R., López-Romero D., Bañuls M.J., Laguna M.F., Barrios C.A., Puchades R., Maquieira A. (2011). Bio-Photonic Sensing Cells over Transparent Substrates for Anti-Gestrinone Antibodies Biosensing. Biosens. Bioelectron..

[24] Grimaldi I.A., Testa G., Persichetti G., Loffredo F., Villani F., Bernini R. (2016). Plasma Functionalization Procedure for Antibody Immobilization for SU-8 Based Sensor. Biosens. Bioelectron..

[25] Murillo A.M.M., Tomé-Amat J., Ramírez Y., Garrido-Arandia M., Valle L.G., Hernández-Ramírez G., Tramarin L., Herreros P., Santamaría B., Díaz-Perales A. (2021). Developing an Optical Interferometric Detection Method Based Biosensor for Detecting Specific SARS-CoV-2 Immunoglobulins in Serum and Saliva, and Their Corresponding ELISA Correlation. Sens. Actuators B Chem..

[26] Maigler M.V., Holgado M., Laguna M.F., Sanza F.J., Santamaria B., Lavin A., Espinosa R.L. (2018). A New Device Based on Interferometric Optical Detection Method for Label-Free Screening of C-Reactive Protein. IEEE Trans. Instrum. Meas..

[27] Holgado M., Maigler M.V., Santamaría B., Hernandez A.L., Lavín A., Laguna M.F., Sanza F.J., Granados D., Casquel R., Portilla J. (2016). Towards Reliable Optical Label-Free Point-of-Care (PoC) Biosensing Devices. Sens. Actuators B Chem..

[28] Murillo A.M.M., Valle L.G., Ramírez Y., Sánchez M.J., Santamaría B., Molina-Roldan E., Ortega-Madueño I., Urcelay E., Tramarin L., Herreros P. (2022). Integration of Multiple Interferometers in Highly Multiplexed Diagnostic KITs to Evaluate Several Biomarkers of COVID-19 in Serum. Biosensors.

[29] Holgado M., Sanza Gutierrez F.J., Laguna Heras M.-F., Lavin Hueros A., Casquel del Campo R. (2015). Interferometric Detection Method.

[30] Gunda N.S.K., Singh M., Norman L., Kaur K., Mitra S.K. (2014). Optimization and Characterization of Biomolecule Immobilization on Silicon Substrates Using (3-Aminopropyl)Triethoxysilane (APTES) and Glutaraldehyde Linker. Appl. Surf. Sci..

[31] Barbero F., Russo L., Vitali M., Piella J., Salvo I., Borrajo M.L., Busquets-Fité M., Grandori R., Bastús N.G., Casals E. (2017). Formation of the Protein Corona: The Interface between Nanoparticles and the Immune System. Semin. Immunol..

[32] Monge Argilés J.A., Blanco Cantó M.A., Leiva Salinas C., Flors L., Muñoz Ruiz C., Sánchez Payá J., Gasparini Berenguer R., Leiva Santana C. (2014). A Comparison of Early Diagnostic Utility of Alzheimer Disease Biomarkers in Magnetic Resonance and Cerebrospinal Fluid. Neurología (Engl. Ed.).

[33] Anbumani S., da Silva A.M., Roggero U.F.S., Silva A.M.P.A., Hernández-Figueroa H.E., Cotta M.A. (2021). Oxygen plasma-enhanced covalent biomolecule immobilization on SU-8 thin films: A stable and homogenous surface biofunctionalization strategy. Appl. Surf. Sci..

[34] Holgado M., Barrios C.A., Ortega F.J., Sanza F.J., Casquel R., Laguna M.F., Bañuls M.J., López-Romero D., Puchades R., Maquieira A. (2010). Label-free biosensing by means of periodic lattices of high aspect ratio SU-8 nano-pillars. Biosens. Bioelectron..

[35] Yang C.C., Chiu M.J., Chen T.F., Chang H.L., Liu B.H., Yang S.Y. (2018). Assay of Plasma Phosphorylated Tau Protein (Threonine 181) and Total Tau Protein in Early-Stage Alzheimer’s Disease. J. Alzheimer’s Dis..

[36] Chen S.D., Huang Y.Y., Shen X.N., Guo Y., Tan L., Dong Q., Yu J.T. (2021). Longitudinal Plasma Phosphorylated Tau 181 Tracks Disease Progression in Alzheimer’s Disease. Transl. Psychiatry.

[37] Ziu I., Laryea E.T., Alashkar F., Wu C.G., Martic S. (2020). A Dip-and-Read Optical Aptasensor for Detection of Tau Protein. Anal. Bioanal. Chem..

[38] Lisi S., Scarano S., Fedeli S., Pascale E., Cicchi S., Ravelet C., Peyrin E., Minunni M. (2017). Toward Sensitive Immuno-Based Detection of Tau Protein by Surface Plasmon Resonance Coupled to Carbon Nanostructures as Signal Amplifiers. Biosens. Bioelectron..

[39] Song C., Deng P., Que L. (2018). Rapid Multiplexed Detection of Beta-Amyloid and Total-Tau as Biomarkers for Alzheimer’s Disease in Cerebrospinal Fluid. Nanomed. Nanotechnol. Biol. Med..

[40] Vestergaard M., Kerman K., Kim D.K., Hiep H.M., Tamiya E. (2008). Detection of Alzheimer’s Tau Protein Using Localised Surface Plasmon Resonance-Based Immunochip. Talanta.

[41] Carlin N., Martic-Milne S. (2018). Anti-Tau Antibodies Based Electrochemical Sensor for Detection of Tau Protein Biomarkers. J. Electrochem. Soc..

[42] Shui B., Tao D., Cheng J., Mei Y., Jaffrezic-Renault N., Guo Z. (2018). A Novel Electrochemical Aptamer-Antibody Sandwich Assay for the Detection of Tau-381 in Human Serum. Analyst.

[43] Dai Y., Chiu L.Y., Chen Y., Qin S., Wu X., Liu C.C. (2019). Neutral Charged Immunosensor Platform for Protein-Based Biomarker Analysis with Enhanced Sensitivity. ACS Sens..

[44] Tao D., Shui B., Gu Y., Cheng J., Zhang W., Jaffrezic-Renault N., Song S., Guo Z. (2019). Development of a Label-Free Electrochemical Aptasensor for the Detection of Tau381 and Its Preliminary Application in AD and Non-AD Patients’ Sera. Biosensors.

[45] Derkus B., Acar Bozkurt P., Tulu M., Emregul K.C., Yucesan C., Emregul E. (2017). Simultaneous Quantification of Myelin Basic Protein and Tau Proteins in Cerebrospinal Fluid and Serum of Multiple Sclerosis Patients Using Nanoimmunosensor. Biosens. Bioelectron..

