# Supplementary material for: A New Optical Interferometric Biosensing System Enhanced with Nanoparticles for Alzheimer’s Disease in Serum
Source: Biosensors (Basel). 2023 Jul 5;13(7):707. doi: 10.3390/bios13070707 (PMC10377685; doi:10.3390/bios13070707)
Supplement: Supplementary file 1 [file biosensors-13-00707-s001.zip › biosensors-2429982-supplementary.pdf]

Table S1. A comparison between different biosensor techniques for tau detection.

Signal transduction	*Analytical method(A)*	*Sensor platform*	*Target*	*Linear detection range*	*Limit of detection(C)*	*Sample tested*	*Label*	*Ref.*
Optical	BLI	Streptavidin interface probe sensor	Tau441	2-55 nM	6.7 nM (8.02 pM)	Buffer and FBS	Label-free	[37]
	Spectrophotometry/ Interferometry	Nanopore sensor with integrated microfluidic network	T-Tau	15.6-2000 pg/mL	15.6 pg/mL in buffer (0.284pM)	Buffer and CSF	Label-free	[38]
	LSPR	Resonance-based immunochip	T-Tau	10-100000 pg/mL	10 pg/mL (0.15 pM)	Buffer	Label-free	[40]
	SPR	Surface plasmon resonance coupled to carbon nanostructures	T-Tau	125-1000 pM	125 pM	Buffer and aCSF(D)	Label-free	[38]
	**Interferometry**	**Two Fabry-Perot interferometers**	**T-Tau**	**12.5 μg/mL to 10 pg/mL in buffer 12.5 μg/mL to 10 pg/mL in serum**	**0.18 pM in buffer 0.18 pM in serum**	**Buffer and Human serum**	**Label-free**	**This work**
Electrochemical	CV and EIS	Anti-Tau antibodies on polycrystalline Au surface	Tau441	10-100 μg/mL	10 μg/mL	Buffer	Label-free	[41]
	DPV	Aptamer-antibody-sandwich	Tau381	0.5-100 pM	0.42 pM in PBS (0.50 pM)	Human serum diluted 1/100	Label-free	[42]
	DPV	Neutral charged immunosensor	T-Tau	0.968-454 pM	0.968 pM	Human serum	Label-free	[43]
	CV and EIS	Aptasensor	Tau381	1-100 pM	0.7 pM	Human serum diluted 1/100	Label-free	[44]
	DVP and EIS	GO/pPG/anti-Tau nanoimmunosensor(B)	T-Tau	0.25-250 nM	150 pM	CSF and human serum	Label-free	[45]
Potentiometric	FET	Transistor-based biosensor	T-Tau	1-10 pM	1 pM in buffer and cell culture media, 10 pM in aCSF	Buffer, cell culture and aCSF	Label-free	[19]

(A) Abbreviations: BLI = Biolayer interferometry; LSPR = Localized surface plasmon resonance; SPR = Surface plasmon resonance; CV = Cyclic voltammetry; EIS = Electrochemical impedance spectroscopy; DPV = Differential pulse voltammetry; FET = Field effect transistor. (B) Abbreviations: GO = Graphene oxide; pPG = poly (propyleneglycol). (C) Conversion to molarity was carried out by assuming a mean tau molecular weight of 55KDa. (D) Abbreviation: aCSF = artificial cerebral spinal fluid.
